# Flavonoids and darkness lower PCD in senescing *Vitis vinifera* suspension cell cultures

**DOI:** 10.1186/s12870-016-0917-y

**Published:** 2016-10-26

**Authors:** Alberto Bertolini, Elisa Petrussa, Sonia Patui, Marco Zancani, Carlo Peresson, Valentino Casolo, Angelo Vianello, Enrico Braidot

**Affiliations:** Department of Agricultural, Food, Animal and Environmental Sciences, University of Udine, via delle Scienze, 91, 33100 Udine, Italy

**Keywords:** Cell cultures, Flavonoids, PCD, Senescence, *Vitis vinifera*

## Abstract

**Background:**

Senescence is a key developmental process occurring during the life cycle of plants that can be induced also by environmental conditions, such as starvation and/or darkness. During senescence, strict control of genes regulates ordered degradation and dismantling events, the most remarkable of which are genetically programmed cell death (PCD) and, in most cases, an upregulation of flavonoid biosynthesis in the presence of light.

Flavonoids are secondary metabolites that play multiple essential roles in development, reproduction and defence of plants, partly due to their well-known antioxidant properties, which could affect also the same cell death machinery. To understand further the effect of endogenously-produced flavonoids and their interplay with different environment (light or dark) conditions, two portions (red and green) of a senescing grapevine callus were used to obtain suspension cell cultures. Red Suspension cell Cultures (RSC) and Green Suspension cell Cultures (GSC) were finally grown under either dark or light conditions for 6 days.

**Results:**

Darkness enhanced cell death (mainly necrosis) in suspension cell culture, when compared to those grown under light condition. Furthermore, RSC with high flavonoid content showed a higher viability compared to GSC and were more protected toward PCD, in accordance to their high content in flavonoids, which might quench ROS, thus limiting the relative signalling cascade. Conversely, PCD was mainly occurring in GSC and further increased by light, as it was shown by cytochrome *c* release and TUNEL assays.

**Conclusions:**

Endogenous flavonoids were shown to be good candidates for exploiting an efficient protection against oxidative stress and PCD induction. Light seemed to be an important environmental factor able to induce PCD, especially in GSC, which lacking of flavonoids were not capable of preventing oxidative damage and signalling leading to senescence.

**Electronic supplementary material:**

The online version of this article (doi:10.1186/s12870-016-0917-y) contains supplementary material, which is available to authorized users.

## Background

Plant senescence is a multifactorial process involving several signalling pathways, which require an active regulation by nuclear genes. The presence of redundant processes confers resilience, which is ensured by the activation of a vicarious pathway when the main process fails to reach completion. In addition, the simultaneous presence of parallel pathways allows modulating and strengthening senescence induction. Three main senescence inducers have been recognized, such as carbon starvation, darkness and developmental process [1]. Starvation and darkness substantially consist in cell sugar depletion, while development is a process more finely regulated by gene activation. Dark treatment, if applied in detached leaves, would not be considered the most appropriate experimental model to mimic the natural process. Hence, other stress phenomena, such as water stress on individual leaves, overlap to the cascade of events leading to cell death [2]. In the case of grape cell cultures, here presented, it is possible to study the effect of darkness avoiding the overlapping of the other stress phenomena mentioned above.

Several hundred genes are expressed during leaf senescence in *Arabidopsis*, some of which are related to flavonoid biosynthesis [3]. On the other hand, those related to flavonoid synthesis could be differentially regulated, depending on the type of senescence, since in cell cultures the developmental- and starvation-induced senescence differs from that induced by darkness [1].

According to this scheme, the hormonal elicitation of plant senescence is specific. Developmental senescence implies the participation of ethylene, methyl jasmonate and salicylate, while the latter is not involved in darkness- and starvation-induced senescence [1]. In particular, by modulating genes, hormones play a crucial role, similarly to what has been found in programmed cell death (PCD) [4]. In this case, the involvement of many signalling molecules and a large interplay network have been described.

In *Arabidopsis* suspension cell cultures, heat-induced cell death and senescence share many similar features of PCD [5], whose involvement has already been described in processes such as hypersensitive reaction (HR), aerenchyma differentiation under hypoxic conditions and xylem differentiation [6]. PCD exhibits peculiar characteristics (i.e. DNA laddering and vacuole fragmentation) that are also found in the late phase of senescence [1].

Flavonoids are widespread secondary metabolites in plants. The most abundant classes are the flavan-3-ols, anthocyanins and flavonols, whereas the most common class of phenolic non-flavonoid antioxidants includes the hydroxycinnamates [7]. Their composition and quality depend on plant growth conditions, geographic location and cultivars.

Anthocyanins and colourless flavonoids are mainly localized in different specialized sub-cellular compartments, such as vacuole and cell wall, where they can reach a higher concentration when compared to the animal counterparts. It is therefore interesting to verify whether their effect might be pro-apoptotic, as it generally occurs in animal cells [8], or anti-apoptotic. Anthocyanin accumulation in pigmented cells can prevent developmental- or oxidative stress-mediated PCD-like death, as seen in lace plant (*Aponogeton madagascariensis*) and *Arabidopsis* cell lines, respectively [9–11]. Pigmented cells, in comparison to non-pigmented ones, are more protected by flavonoids against the oxidative stress [12]. This suggests that these metabolites possess an anti-apoptotic effect, related to a decrease in reactive oxygen species (ROS) production and propagation. Hence, the anti-apoptotic mechanism proposed for flavonoids in plants could be generally rationalised as an anti-oxidant activity. This effect could also explain the delay of ripening and senescence, reported in anthocyanin-enriched varieties of tomatoes [13].

Anthocyanins and colourless flavonoids also perform a key role in human health, acting as antioxidants by preventing some ROS-associated diseases, such as cancer [14, 15], or acting as tumour-inhibiting natural molecules in cancer cell lines [16–19].

Grapevine (*Vitis vinifera* L.) is a widespread cultivated plant rich in polyphenols (mainly flavonoids and stilbenes), which are present in most tissues. They are synthesized and accumulated during the plant cycle and play several roles in response to biotic and abiotic stress. Grapevine flavonoids, including anthocyanins, are powerful antioxidants, protecting leaves and berries against UV photo-oxidative damage, but could also act as seed dispersers or pollinator recruiters [20, 21]. In grapevine cell cultures, treatment with cellulase elicits HR-like responses, causing localised cell death, browning and inducing phenolic metabolism [22]. In accordance, Repka and co-authors showed that the HR, elicited by methyl jasmonate in grapevine, induces the activation of genes related to defence, PCD and phenylpropanoid biosynthesis [23, 24]. Nevertheless, in all these studies on plant elicitor- or apoptosis activator-induction of PCD, it is difficult to distinguish whether flavonoid accumulation in the cell is among the consequences of HR, or the main cause of cell death through induction of a pro-apoptotic effect.

In the present work, starving solid grapevine cell cultures grown under light were obtained by extending their growth largely beyond their proliferation rate plateau. This growth condition induced a pigmented flavonoid production on the outer layers of the cell aggregates, whereas the inner ones, close to nutrients of the medium and protected from an excess of light, remain green and not pigmented. Red and green cells were obtained from this different material and separately sub-cultured in liquid medium under different light regimes (light vs. darkness). These suspension cell cultures represent a simple model-system to study the role of endogenous flavonoids in senescence and PCD, avoiding multi-factorial interactions between flavonoid biosynthesis induction and senescence modulation (i.e. osmotic stress, hormonal concentration, nutrient availability, ROS or oxidative stressors). Therefore, this paper focuses on the main effects exerted by dark and light conditions on grapevine senescing suspension cell cultures. In addition, endogenous flavonoids were considered with respect to the role they could play in senescence and/or PCD.

## Methods

### Plant material and cell cultures on solid medium

Long-term callus cultures of grapevine (*Vitis vinifera* L., cv. Limberger), established from young leaf tissues, were kindly supplied by V. Repka, Research Institute of Viticulture and Enology, Bratislava, Slovakia. The calli were then maintained on solid media under light, according to Repka et al. [25], with minor modifications. Their maintenance was obtained by sub-culturing them every 14 days for several cycles. For the experiments in suspension cell cultures, this period was extended to 23 days, 1 week longer than the usual growing cycle. At this stage, cells had reached a stationary phase of growth, underwent nutrient starvation and, therefore, had triggered the pathway leading to senescence. The pigmented red cells from 23-day-old cultures, localised on the external cell layers, were selected as a starting inoculum for obtaining Red Suspension cell Cultures (RSC). Similarly, the lightly-green cells in the underneath layers were chosen for obtaining Green Suspension cell Cultures (GSC). RSC and GSC were used for experiments after 3 and 6 days after subculture, in the presence of either darkness or light, respectively.

At day 0, both GSC and RSC were also analysed for the content of soluble hexoses (glucose and fructose, respectively) and for total protein concentration, as general hallmarks of the senescence programme [26, 27]. Aliquots of 100 ± 20 mg FW from 3 independent calli were ground into frozen powder under liquid nitrogen. For protein analysis, the powder was resuspended in 0.5 ml of 50 mM Tris-HCl (pH 7.5) and protein measured by Bradford method [28] and expressed as mg g^−1^ FW.

For sugar analysis, the powder was suspended in 1 ml of 50 mM Tris-HCl, 0.05 % Triton X-100, boiled at 100 °C for 5 min and centrifuged by a Mikro 120 Hettich centrifuge. The supernatant was used for the standard enzymatic assay of glucose and fructose, measured by a Perkin-Elmer fluorimeter at the wavelengths of 329 and 460 nm for excitation and emission, respectively [29]. Hexose content was expressed as μmol g^−1^ FW.

### Suspension cell cultures

For all experiments, 4 g (fresh weight, FW) of either GSC or RSC were transferred into 120 ml of fresh liquid medium contained in 250 cm^3^ Erlenmeyer flask. The flasks were maintained in an INNOVA 2300 platform shaker, rotating at 110 rpm and kept at 27 ± 1 °C. Finally, GSC and RSC were grown for 6 days either under light (12 h light period) or in dark conditions, respectively.

### Determination of growth rate

Aiming at estimating the growth rate of cell cultures grown on solid medium, 3 g FW of callus pieces was initially transferred to a fresh solid medium. Calli were picked up and re-weighed after 3, 6, 9, 12, 15, 18, 21 and 23 days of proliferation.

### Extraction of flavonoids

Grapevine GSC and RSC were grown under light conditions, harvested at day 0 and 6, frozen in liquid nitrogen and ground to a fine powder. Then, 0.5 g (FW) of frozen powder was incubated overnight with 1 ml of 15 % (*v/v*) HCl in methanol to extract flavonoids. The alcoholic extracts (suspensions) were then centrifuged 10 min at 12,000 *g* in a Mikro 120 Hettich centrifuge. The supernatants were recovered, dried under N_2_ flow and finally dissolved in 200 μl of mobile phase (85 % methanol and 15 % bidistilled water).

### RP-HPLC analysis of flavonoids

HPLC separation and quantification were performed with 0.5 % (*v/v*) acetic acid, pH 2.5 (eluent A) and methanol (eluent B) at a flow rate of 0.5 ml min^−1^. RP-HPLC was performed on a Zorbax Eclipse extra dense bonding-C18 column (5 μm, 4.6 × 150 mm, Agilent 1200 series Instrument), equipped with a binary pump delivery system, coupled to a DAD. The binary gradient conditions were: 27–44.5 % B (32 min), then 44.5–67.5 % B (13 min), 67.5–100 % B (2 min), isocratic 100 % B (3 min). Anthocyanins were monitored at 520 nm and identified by comparison of their chromatographic profile with the commercial standard malvidin 3-O-glucoside (Extrasynthese, Lyon France).

### Mass spectrometry of alcoholic extracts

Aiming at identifying the main polyphenolic compounds shown by HPLC analysis, alcoholic extracts were obtained as described above from GSC and RSC grown under light for 6 days, and then used for mass spectrometry characterization. Multi-stage Mass Spectrometry (MS^n^) experiments and liquid chromatography - electrospray ionization - mass spectrometry (LC-ESI-MS) analyses were achieved by a Finnigan LXQ Linear Ion Trap, operating in the negative ion mode, coupled with a Dionex UltiMate 3000 RS Pump and equipped with a Dionex UltiMate RS 3000 Autosampler (Thermo Scientific, San Jose, CA, USA). Methanolic standard solutions (200 μg ml^−1^) of gallic acid, quercetin glucoside, quercetin, malvidin-glucoside and kaempferol were infused into the ion source at a flow rate of 10 μl min^−1^, with the aid of a syringe pump, in order to obtain the corresponding MS^n^ spectra (see Supplementary material). Only in the case of cyanidin-glucoside, the identification was performed using data retrieved from an *in silico* library (http://www.massbank.jp/jsp/Dispatcher.jsp?type=disp&id=PR020036&site=1).

The typical source conditions were: transfer line capillary at 275 °C, ion spray voltage at 4.70 kV, sheath, auxiliary and sweep gas (N_2_) flow rates at 20, 5 and 0 arbitrary units, respectively. Helium was used as the collision damping gas in the ion trap, set at a pressure of 1 mTorr. ESI-MS^n^ spectra were obtained by collision induced dissociation (CID) experiments, after isolation of the appropriate precursor ion in the ion trap (isolation width 1.2 *m/z* unit), and subjecting them to the following typical conditions: normalized collision energy between 20 and 30 %, selected to preserve a signal of the precursor ion in the order of 5 %; activation *Q* 0.25 and activation time 30 ms. The HPLC separations were performed on a Synergi 4 μm Hydro-RP 80A column (250 mm × 2.0 mm) from Phenomenex (Bologna, Italy). The mobile phase consisted of 0.2 % (*v/v*) formic acid in water (eluent A) and 0.2 % (*v/v*) formic acid in methanol (eluent B), and the linear gradient elution conditions were as follows: 27–44.5 % B (32 min), 44.5–67.5 % B (13 min), 67.5–100 % B (2 min), 100 % B isocratic (19 min), 100-27 % B (3 min), 27 % B isocratic (6 min) at a constant flow rate of 0.1 ml min^−1^ at 30 °C.

### Determination of dead cells

GSC and RSC were incubated with a solution containing fluorescein diacetate (FDA) to determine cell viability. The FDA working solution was prepared as described by McCabe and Leaver [30]. Cytochemically-stained cells were observed under a LEICA Fluovert fluorescence microscope, using a Nageotte chamber, counting at least 100 aggregate-free cells. The percentage of dead cells was calculated by considering the ratio between the FDA-unstained and the total number of cells.

### Reactive oxygen species (ROS) determination

The generation of ROS was monitored according to the methods of Ledoux et al. and Santos et al. [31, 32], using 2′, 7′-dichlorodihydrofluorescein diacetate (H_2_DCFDA) as a probe. Samples of suspension cell cultures (2 ml), at day 0, were incubated in 24-well cell culture cluster with 5 μM H_2_DCFDA. The detection was performed for 105 min using Multilabel Counter (WALLAC, model 1420, Perkin-Elmer) with 5 min-intervals readings, using excitation at 485 ± 10 nm and emission at 535 ± 10 nm. GSC and RSC were also incubated with 1 mM 2,2-azobis(2-methylpropionamide)hydrochloride (ABAP) as free radical initiator. The extent of cell death was evaluated at the beginning and at the end of the assay.

### Determination of cellular ATP

GSC and RSC (approx. 10 g FW), at different sampling days (0, 3 and 6), were first filtered by nylon gauze (100 μm mesh), then frozen with liquid nitrogen and finally ground to a fine powder. Samples (100 ± 20 mg FW) were re-suspended in 1 ml of 50 mM Tris–HCl (pH 7.5) and 0.05 % (*w/v*) Triton X-100, and immediately boiled for 2 min. After centrifugation (10 min at 12,000 *g* in a Mikro 120 Hettich centrifuge), aliquots of supernatant were used for luminometric assay, as described by Petrussa et al. [29]. ATP calibration curve was performed for each experiment and the sample concentrations were then calculated by interpolation. Apoptotic or necrotic samples at day 0 were prepared as a control by incubating either in ethanol 10 % (*v/v*) for 24 h or at 80 °C for 10 min, respectively.

### Detection of cytochrome *c* release

Approximately 4 g FW of GSC or RSC, grown under either light or dark conditions for 6 days, was used for the cytosolic extracts. The material was homogenised with 4 ml of homogenisation buffer (20 mM HEPES-Tris, pH 7.6; 0.3 M sucrose; 1 mM EDTA; 5 mM DTE; 2 mM PMSF; 1 mM benzamidine and 0.6 % (*w/v*) PVPP), filtered through a nylon gauze (100 μm mesh) and then centrifuged at 1000 *g* for 10 min at 4 °C by Mikro 120 Hettich centrifuge, to eliminate debris. The supernatant was centrifuged again at 15,000 *g* for 20 min. The obtained supernatant was further ultracentrifuged at 100,000 *g* for 40 min by a Beckman L7-55 centrifuge (Ty 70ti rotor) to obtain the final soluble fraction. Soluble proteins were concentrated by 5000 MWCO concentrators VIVASPIN 4 (Sartorius, Göettingen, Germany) at 10,000 *g* for 30 min (Mikro 120 Hettich centrifuge). Soluble proteins (ca. 30–40 μg) were separated by 15 % (*w/v*) SDS-PAGE and electroblotted onto a nitrocellulose membrane. The blots were incubated at 4 °C overnight with 200 μl of a polyclonal anti-cytochrome *c* Ab (Agrisera), at 1/1000 dilution. The cross-reaction was finally detected by nitroblue tetrazolium and 5-bromo-4-chloro-3-indolyl phosphate colour development, after incubation with alkaline phosphatase-conjugated anti-rabbit IgG antibody (1/2500 dilution; Sigma, St. Louis, MO, USA). Computer-assisted densitometric analysis of immunoblots was quantified using Quantity One software (Bio-Rad, Hercules, USA).

### *In situ* detection of DNA fragmentation (TUNEL assay)

Samples of GSC and RSC (1 ml suspension) were collected at day 0 and 6 and washed three times in phosphate buffered saline (PBS), incubated with fixation solution of 2 % (*v/v*) paraformaldehyde in PBS for 60 min. Then, samples were treated with permeabilisation solution (0.1 % (*w/v*) Triton X-100 in 0.1 % (*w/v*) sodium citrate) and washed three times with PBS. Samples were labelled with TUNEL reaction mixture (TMR-red *in situ* cell death detection kit, Roche Diagnostics) in darkness at 37 °C for 60 min. Negative (without terminal transferase) and positive (with ethanol treatment at day 0, as described by Hogg et al. [33]) samples were properly included. For nuclear staining, the samples were washed twice by PBS and stained with 1 μg ml^−1^ 4′,6-diamidino-2-phenylindole (DAPI) for 15 min. Finally, all samples were examined under a Leitz Fluovert fluorescence microscope, with two sets of filters: 360 nm excitation and 420 nm emission for DAPI detection; 540 nm excitation and 620 nm emission for TUNEL, respectively. The percentage of apoptotic TUNEL-positive nuclei was determined by counting at least 200 nuclei.

### Statistical data analysis

Treatment group means were compared by LSD (Least Significant Difference), according to Fisher’s statistical test, and different letters, assigned to means, designate a statistical difference at *P* ≤ 0.05. Since cell death and TUNEL positive cells were evaluated as percentage on the total amount of cells, the statistical treatment was performed on such data after their transformation by the formula arcsen (x^1/2^). A *t*-test was applied for comparison of protein and sugar content among GSC and RSC calli at day 0.

## Results

### Production of suspension cell cultures from *V. vinifera* (cv. Limberger) and characterization of their flavonoid profile

Grapevine cell cultures were grown under light on a solid medium. After 14 days, these cells reached the optimal growth phase for sub-culturing on solid medium. The growth exhibited a sigmoidal trend that reached a steady-state from 21 to 23 days (Additional file 1: Figure S1). At this stage, accumulation of red pigments on the surface of calli was detected (result not shown). Therefore, red (pigmented) and green (non-pigmented) clusters from the same callus piece were chosen as inoculum for the subculture into liquid medium in order to study the effects of endogenous flavonoid at the onset of senescence. As specified in the Methods section, cell types were obtained from the same callus piece and differed by their relative position (internal/green or external/red). Moreover, green and red cells did not significantly differed in their glucose and fructose concentration (Additional file 2: Table S1), suggesting that both cell types were subjected to a similar degree of senescence induction by hexose signalling. The difference in total protein content, as hallmark of cell degradation, was statistically significant among the cell types (*p* = 0.01). However, the decrease in protein was noticeable in red cells, which showed to be even more protected from PCD than green ones, during subsequent transfer in liquid culture.

**Fig. 1:**
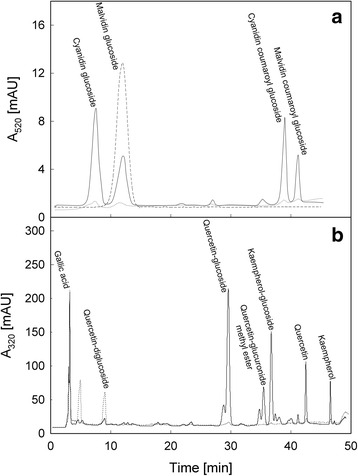
RP-HPLC analysis of anthocyanins (**a**) and polyphenolic profile (**b**) from alcoholic extracts obtained by *V. vinifera* (cv. Limberger) suspension cell cultures. GSC (*dotted line*) and RSC (*solid line*) were grown under light conditions for 6 days. Their metabolite content was determined at day 6. Chromatographic profiles of anthocyanidin glucosides, cyanidin and malvidin, as well as their respective substituted derivatives, are presented. Malvidin glucoside (*dashed line*) was used as a standard (Panel **a**). The identification was obtained by mass spectrometry analysis on chromatographic peaks detected at 520 nm. Similar analysis was performed at 320 nm (Panel **b**) and the retrieved flavonoids were identified as follows: 1) gallic acid; 2) quercetin-diglucoside; 3) quercetin-glucoside; 4) quercetin-glucuronide methyl ester; 5) kaempferol-glucoside; 6) quercetin; 7) kaempferol. Data are representative of three different experiments

The two suspension cell cultures (GSC and RSC) were then maintained in a liquid medium for 6 days under light or darkness. Since the treatments lasted just 6 days, it was considered that after this period dark- and light-treated cell cultures did not substantially differ for their flavonoid content. For this reason, only in light-grown RSC and GSC, anthocyanin content and composition were analysed by RP-HPLC (Fig. 1, Panel a). The chromatograms show that Limberger cells accumulated mainly cyanidin and malvidin glucosides, as well as their coumaroyl derivatives. In particular, anthocyanin content in GSC was low, with a similar pattern at day 0 (data not shown) and 6 (Panel a, dashed line), respectively, demonstrating that light treatment did not change their flavonoid concentration or composition after 6 days. Conversely, RSC showed an appreciable amount of anthocyanins after 6 days (Panel a, solid line), when compared to GSC. To characterize further the different classes of flavonoids accumulated in both suspension cell cultures at day 6, mass spectrometry (MS) analysis was also performed in alcoholic extracts (Additional file 3: Figure S2). The HPLC chromatograms revealed that, besides to anthocyanins, RSC mainly accumulated gallic acid, quercetin-glucoside, quercetin, kaempferol-glucoside and kaempferol (Fig. 1, Panel b, solid line). GSC synthesized mainly gallic acid and quercetin-diglucoside (Fig. 1, Panel b, dotted line). The presence of kaempferol-hexose ([M-H]ˉ ion at *m/z* 447), kaempferol-dihexose ([M-H]ˉ ion at *m/z* 609), quercetin-dihexose ([M-H]ˉ ion at *m/z* 625) and quercetin-glucuronide methyl ester ([M-H]ˉ ion at *m/z* 491) was shown by the mass spectrum of cell extracts obtained by infusion. The identification of these compounds is based on their MS^n^ fragmentation behaviour (Additional file 4: Figure S3, Additional file 5: Figure S4, Additional file 6: Figure S5 and Additional file 7: Figure S6). In particular, these compounds show the loss of the hexose (162 Da) or glucuronide methyl ester (190 Da) moieties, generating the corresponding glycoside ions at *m/z* 285 and 301 that, upon further fragmentation, exhibit the typical fragments of kaempferol and quercetin, respectively.

### Effects of darkness and endogenous flavonoids on viability of suspension cell cultures

**Fig. 2:**
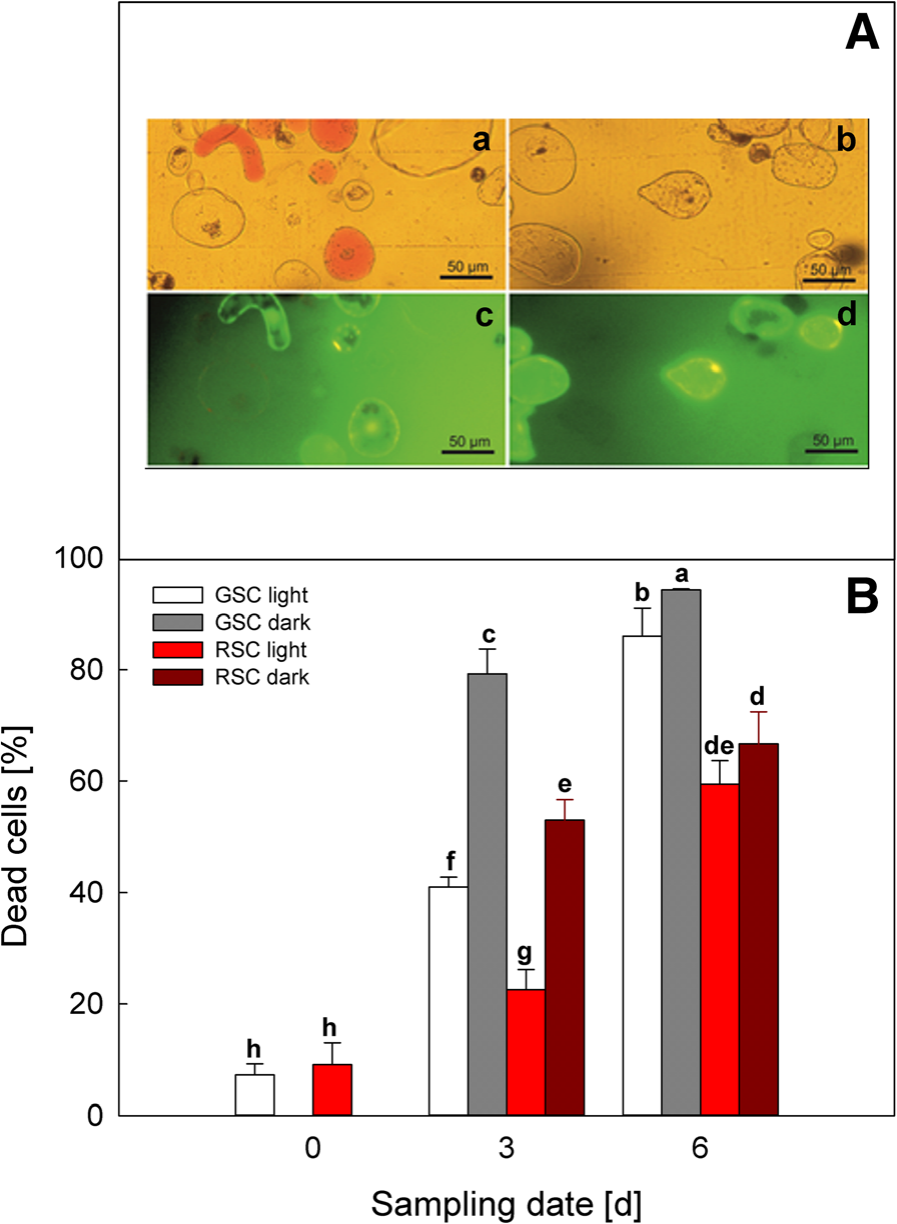
Cell death in *V. vinifera* (cv. Limberger) suspension cell cultures evaluated by FDA staining. Panel **a** GSC (*b*, *d*) and RSC (*a*, *c*) were grown under light conditions for 6 days. After staining with FDA, they were analyzed under fluorescent (*c*, *d*) or visible (*a*, *b*) light. Panel **b** time-course of total cell death in GSC and RSC, grown under light or dark conditions, and sampled at day 0, 3 and 6, respectively. The percentage of dead cells was calculated by the ratio between FDA-stained and total cell number. *Bars* are means ± S.D. of at least three different experiments. Different letters indicate a significant difference (*P* ≤ 0.05), evaluated by ANOVA test

Light treatment was used as a control to determine the possible effects of darkness and flavonoids on cell viability. Staining with FDA was employed to evaluate the viability of the different cell cultures. Figure 2 (Panel a) shows the morphological features at day 6 of RSC (*a*) and GSC (*b*) under visible light, whereas in FDA-stained samples (*c* and *d*), only the alive fluorescent cells were counted. Cell death increased during the whole experimental period, reaching a maximum at day 6 in all samples (Panel b).

This trend was higher in GSC and it was stimulated by darkness, albeit this effect was less evident at day 6. Even in this case, the difference between light- and dark-treated cells was lower at day 6.

### Characterization of PCD markers in suspension cell cultures

Different biochemical and molecular markers of PCD were analysed in order to determine: i) if the observed cell death in the two suspension cell cultures grown under light or dark conditions showed PCD hallmarks; ii) how flavonoids could modulate the PCD manifestation.

**Fig. 3:**
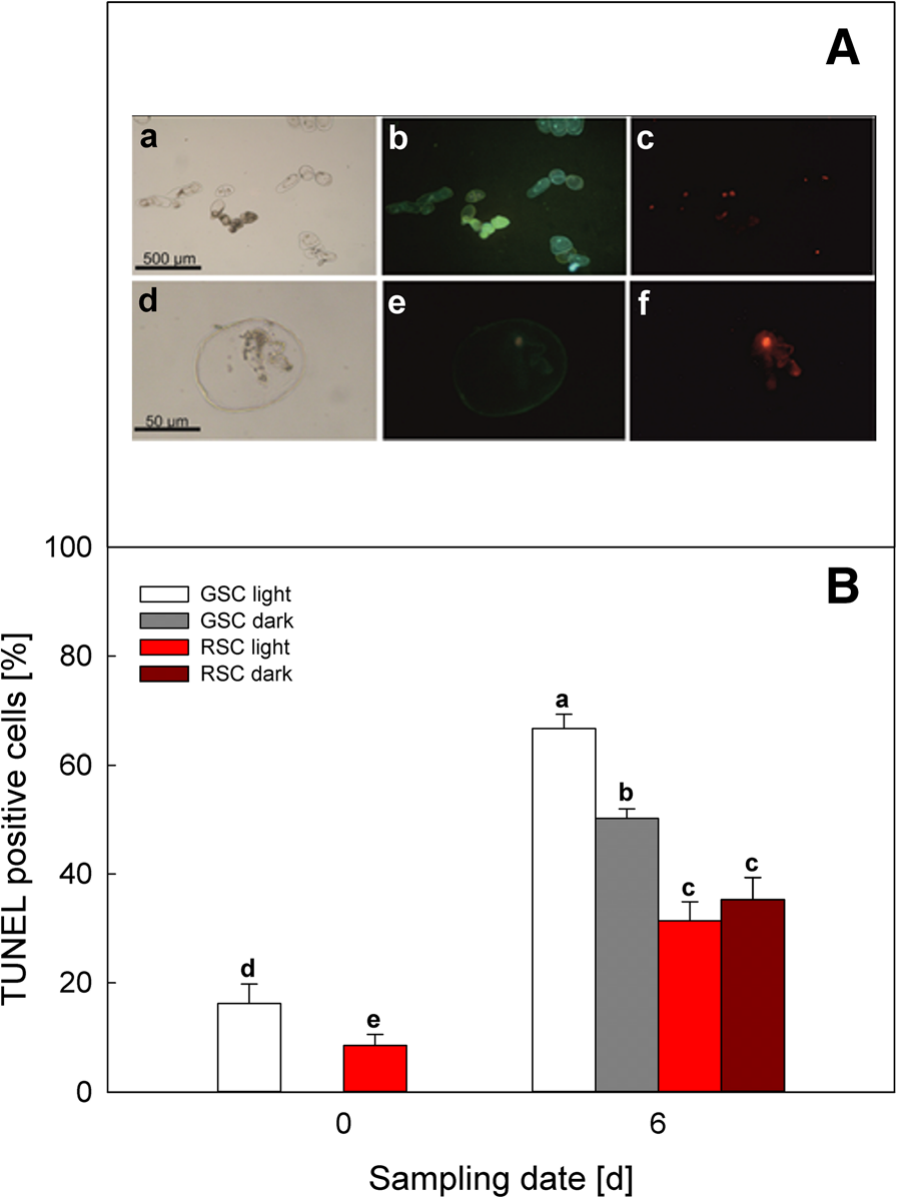
PCD in *V. vinifera* (cv. Limberger) suspension cell cultures, evaluated by TUNEL assay. Cells at day 0 were observed under visible light (*a*, *d*); nuclei were stained with DAPI (*b*, *e*) or TMR-red for TUNEL assay (*c*, *f*), and observed under UV light, with low (*b*, *c*) and high (*e*, *f*) magnification (Panel **a**). TUNEL assay was performed in GSC and RSC grown under light or dark conditions at day 0 and 6, to evaluate the percentage of cells undergoing PCD (Panel **b**), counting the cells with red fluorescent-stained nuclei as apoptotic-like dead cells. *Bars* are means ± S.D. of at least three different experiments. Different letters indicate significant difference (*P* ≤ 0.05), evaluated by ANOVA test

TUNEL and DAPI assays were performed on all samples at day 0 and 6 (Fig. 3). Panel a shows the feature of the cells, which were positive to the DAPI and TUNEL reaction. The blue fluorescence-stained nuclei (*b*, *e*) were counted as an estimation of the total cell number, while TMR red-fluorescent nuclei (*c*, *f*) represented fragmented nuclei in PCD-undergoing cells. These parameters allowed us to estimate the amount of cells undergoing PCD in different treatments at day 0 and 6 (Panel b), because just these samples showed the highest differences in ATP levels between GSC and RSC (see later). PCD was higher in GSC, whereas darkness inhibited it. In RSC, flavonoids significantly decreased the amount of TUNEL positive cells and also abolished the effect of darkness, showing that light stimulated PCD only in GSC.

**Fig. 4:**
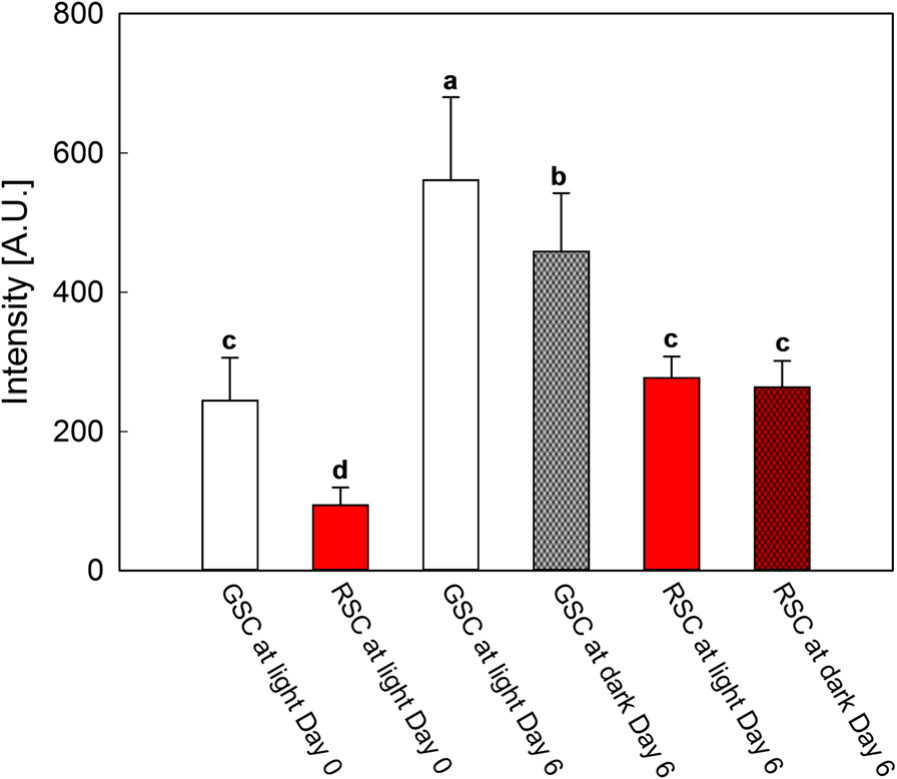
Cytochrome *c* release in cytosolic fractions isolated from *V. vinifera* (cv. Limberger) suspension cell cultures. GSC and RSC were grown either under light or dark conditions for 6 days. Samples were obtained at day 0 and 6, respectively, for the analysis of cytochrome *c* release. The densitometric analysis of cross-reactivity signals were detected after Western blot of cytosolic proteins isolated from cell cultures, incubated with anti-cytochrome *c* primary antibody. *Bars* are means ± S.D. of at least three independent experiments. Different letters indicate significant difference (*P* ≤ 0.05), evaluated by ANOVA test

Another hallmark of PCD is represented by cytochrome *c* release from mitochondria (Fig. 4). This analysis was performed on cytosolic fractions from different treatments at day 6, in comparison to day 0, as a control. The densitometric analysis of the antibody cross-reaction showed that cytochrome *c* release was higher in GSC, in particular under light, than in RSC, in agreement with the results on TUNEL assay. Darkness partially inhibited this release, but had no effect in RSC, in accordance to what was observed in TUNEL analysis.

**Fig. 5:**
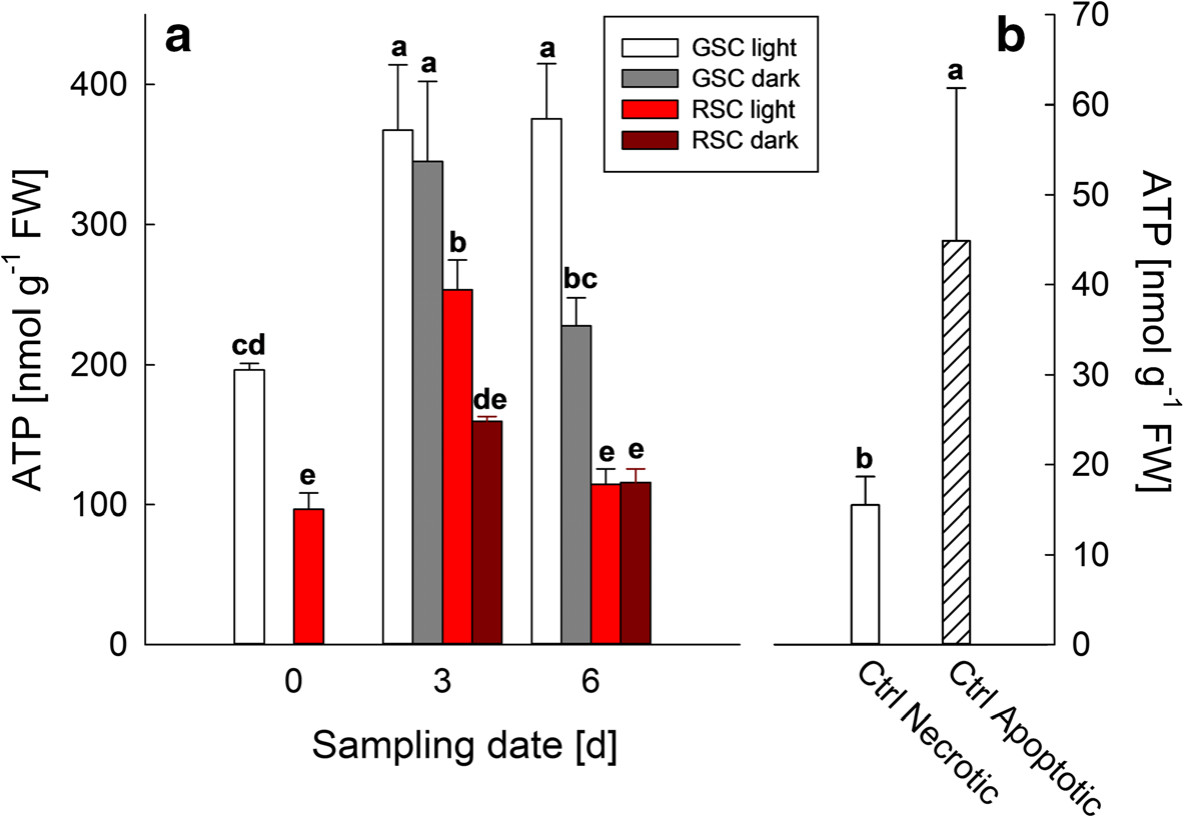
ATP content in *V. vinifera* (cv. Limberger) suspension cell cultures. GSC and RSC were grown under light or dark conditions for 6 days, and sampled at day 0, 3 and 6, respectively (Panel **a**). Necrotic- and apoptotic-like samples, used as positive controls, were obtained on GSC at day 0, after incubation at 80 °C for 10 min or with 10 % (*v/v*) ethanol for 24 h, respectively (Panel **b**). *Bars* are means ± S.D. of at least three independent experiments. Different letters indicate significant difference (*P* ≤ 0.05), evaluated by ANOVA test

Since ATP level is known to be crucial for the progress of PCD, its cellular level was assessed at different sampling dates (0, 3 and 6 days, Fig. 5, Panel a). At day 0, a significant difference between GSC and RSC was observed; in particular, the highest ATP concentration detected in GSC under light could be ascribed to a higher photosynthetic activity. Moreover, ATP content in light-treated GSC increased from day 0 to day 3 and was comparable at day 6, while in dark conditions GSC underwent a fall in ATP concentration at a level similar to the initial one. In RSC under light treatment ATP level reached its maximum at day 3 and, thereafter, declined at day 6, while in dark conditions it remained constant at both day 3 and 6. To evaluate the level of ATP associated to PCD manifestation, a positive control was performed by treating GSC at day 0 with 10 % ethanol (83 ± 0.11 % of PCD, data not shown), in comparison to treatment at 80 °C for 10 min (100 % necrotic cells, data not shown), (Panel b).

### Reactive oxygen species (ROS) generation in suspension cell cultures

**Fig. 6:**
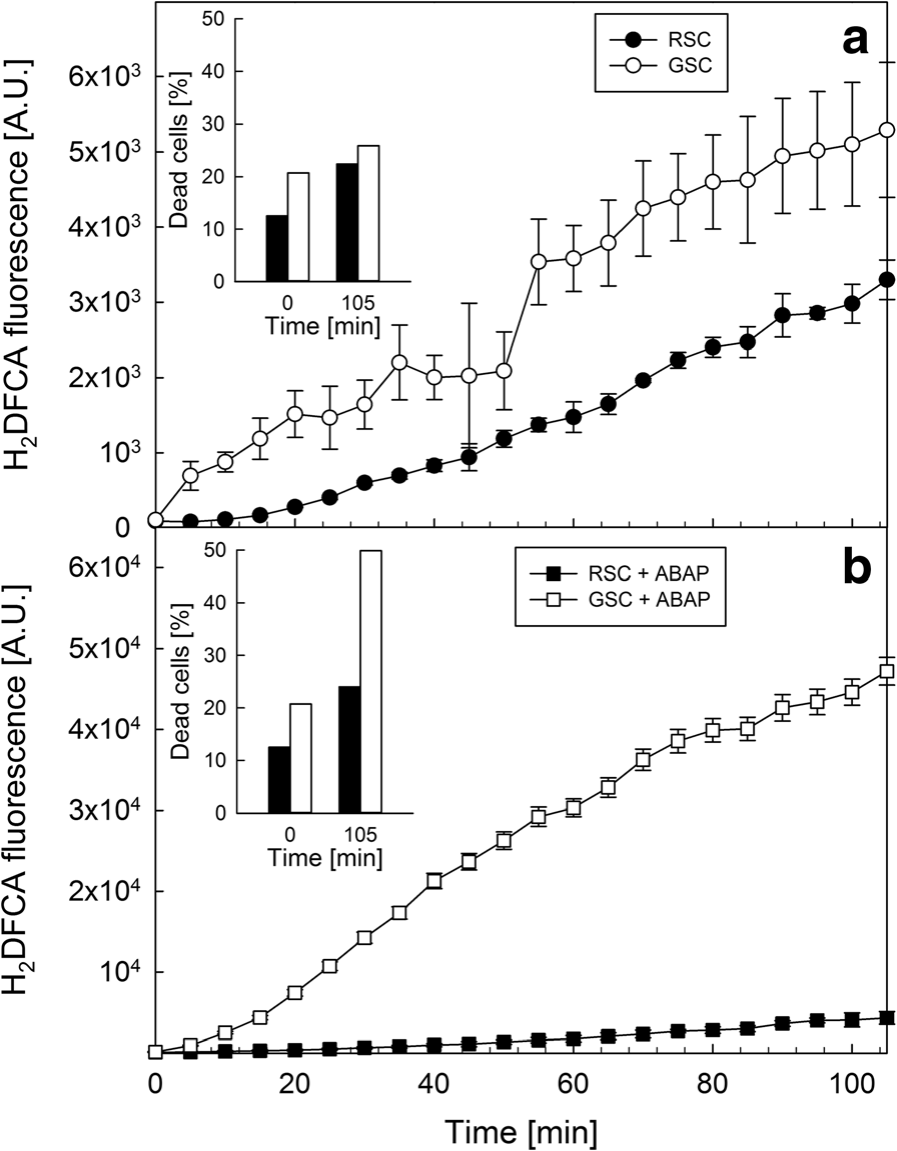
Reactive oxygen species (ROS) formation in *V. vinifera* (cv. Limberger) suspension cell cultures. ROS generation was estimated as fluorescence intensity generated by H_2_DFCA in GSC and RSC at day 0. Cell cultures were incubated in the absence (Panel **a**) or presence (Panel **b**) of 1 mM ABAP. *Insets* represent the total amount of dead cells, evaluated by FDA staining at either 0 and 105 min. *Bars* are means ± S.D. of at least three independent experiments

Since at day 0 GSC and RSC exhibited comparable levels of cell death, it was interesting to assess if the level of ROS production was different in the two systems. Both cell cultures were incubated with the fluorescent probe H_2_DCFDA, to monitor the time-course of ROS production (Fig. 6). Panel a shows that GSC generated a significant higher amount of ROS in comparison to RSC, but this ROS overproduction did not induce appreciable differences in cell death after 105 min (inset Panel a). On the contrary, when cells were treated with the free radical generator ABAP, GSC exhibited a dramatic increase of ROS production (Panel b), paralleled by a strong increase of cell death (inset Panel b), which was not observed in RSC.

## Discussion

Flavonoids play several and relevant physiological roles. First, they act as antioxidants, preventing cell death by delaying/inhibiting the activation of genes related to PCD. However, flavonoids need to be constantly reduced in order to function as antioxidants. This could be achieved only by an efficient reducing system, such as that of ascorbate and reduced glutathione, which are supplied by photosynthesis. Second, flavonoids may cause mild uncoupling of the mitochondrial oxidative phosphorylation, inducing a higher electron flux through the respiratory chain and decrease of transmembrane potential, thus preventing ROS generation by mitochondria [34].

In the present work, the effect of darkness and flavonoids in senescence was assessed by utilizing two grapevine cell cultures obtained from the same callus, one with a high (RSC) and the other with a low (GSC) content of these secondary metabolites (Fig. 1). The starting material grown on solid medium was prone to senescence due to nutrient starvation, whereas the onset of pigmentation observed on the surface of callus was mainly caused by an excess of light on these cells. When sub-cultured in liquid medium, their difference in secondary metabolites at day 0 was, nevertheless, associated to a similar level of cell death. Since GSC and RSC possessed the same genotype, as they were originated by the same callus, they did not genetically differ between each other, except for a stimulated flavonoid biosynthesis pathway and for a different capacity of scavenging ROS overproduction (Fig. 6, Panel b).

The level of cell death in GSC and RSC was low at the beginning of subculture (day 0) and substantially comparable, but after 3 days in liquid medium, dark conditions strongly stimulated cell death in both cell cultures (Fig. 2). Such a behaviour is in agreement with previous results [35, 36], demonstrating that darkness is an inducing factor of cell death. This effect was more evident at day 3 rather than at day 6 (Fig. 2). The presence of flavonoids decreased this effect in both dark- and light-treated cells.

Buchanan-Wollaston et al. [1] have shown that senescence induced by darkness or starvation exhibits a distinct transcriptome pattern, if compared to the developmental program. In addition, darkness increases ROS production [37]. Accordingly, at day 0 (after 23 days of starvation on solid medium), GSC showed higher ROS evolution than RSC (Fig. 6), because flavonoids, acting as antioxidants, decreased ROS production.

After 6 days, apoptosis-like PCD became apparent, as shown by TUNEL assay (Fig. 3) and cytochrome *c* release (Fig. 4). Apoptosis-like PCD was more pronounced in light-grown GSC than in those grown in the darkness. Flavonoids decreased this type of death, confirming that, in addition to dark-induced cell death, these metabolites specifically protected the cells against PCD. Indeed, a high ATP level, which favours the execution of apoptosis [38, 39], was still evident after 6 days in GSC and was compatible with the occurrence of apoptosis-like PCD (Fig. 5, Panel a). The higher content of ATP in GSC, if compared to RSC, can be explained with a more efficient photosynthetic activity in the former cells, which was already evident at day 0. The difference in ATP content between GSC and RSC was amplified also by light treatment, because only suspension cell cultures grown in light have functional chloroplasts [40]. Darkness lowered ATP in both GSC and RSC, albeit this effect was more pronounced in the RSC after 6 days.

The protecting effect of flavonoids against cell death, particularly after 6 days, could be due to their antioxidant activity [41, 42]. ROS could be therefore maintained below a critical level to avoid that they become cytotoxic and thus acting as necrosis inducers [43]. This protection could even prevent ROS from behaving as signals to trigger apoptosis-like PCD [44] in RSC mitochondria, where ROS synthesis is known to be lower, if compared to that of chloroplast [45].

It should be stressed that the effect of plastidial ROS has been found to occur downstream of the mitochondrial release of pro-apoptotic factors, but before the caspase activation [46–48]. In GSC grown in the light additional PCD could thus be induced, by overlapping of mitochondrial and chloroplast pathways. The apoptosis-like PCD, occurring mainly in light-grown GSC (Figs. 3 and 4), could also depend on the involvement of chloroplasts [44, 49]. In agreement to this, in *Arabidopsis* UV light alone induces PCD mediated by caspase-like activities [50]. This could explain the increase in PCD that was observed in light-treated GSC even if mitochondrial pathway was predominant. Conversely, in light- and dark-treated RSC, flavonoids protected from PCD (Figs. 3 and 4), which is a phenomenon particularly evident at day 6.

## Conclusions

In conclusion, darkness was responsible for triggering mainly necrotic cell death, induced by high ROS production, coupled with an inefficient antioxidant system. It is noteworthy that cells transferred from solid to liquid media had to face a strong oxidative environment. Conversely, PCD was more stimulated in light-grown GSC, since the ROS triggering was not counteracted by flavonoids. The involvement of polyphenolic compounds may be also hypothesized at mitochondrial level, as already suggested in mammals, where such compounds decrease hydrogen peroxide formation by interacting with complex I [34]. Similarly, these secondary metabolites could minimize the oxidative damage caused by plastidial ROS.

### Supplementary material

Additional file 1: Figure S1 was added as supplementary material and shows proliferation rate of *V. vinifera* (cv. Limberger) cell cultures grown on solid medium. Additional file 3: Figure S2, Additional file 4: Figure S3, Additional file 5: Figure S4, Additional file 6: Figure S5 and Additional file 7: Figure S6 were also added as supplementary materials and show the MS spectra regarding grapevine cell culture alcoholic extracts and reference standards.

Additional file 2: Table S1 was added as supplementary material and shows protein and hexose content of cell cultures at day 0.

## Additional files

Additional file 1: Figure S1. Proliferation rate of *V. vinifera* (cv. Limberger) cell cultures grown on solid medium. Cell cultures were grown under light for 23 days. Bars represent means ± S.D. of at least three independent experiments. (TIF 176 kb)
Additional file 2: Table S1. Senescence hallmarks in GSC and RSC. Measurement of total protein content and hexose concentration in GSC and RSC at day 0, just before liquid culture establishment. (DOCX 13 kb)
Additional file 3: Figure S2. Mass spectra obtained using Multi-stage Mass Spectrometry (MS^n^) for analysis of alcoholic extracts obtained by *V. vinifera* (cv. Limberger) suspension cell cultures. Upper spectrum represents the spectrum obtained from crude extract obtained from RSC grown for 6 days under light. Middle spectrum and lower spectrum represent spectra from two different fractions eluted with H_2_O and methanol from C18 SPE column, respectively. (TIF 47 kb)
Additional file 4: Figure S3. Structural characterization of malvidin-glucoside standard by Mass spectra, using MS^n^. Upper spectrum shows full spectrum of malvidin-glucoside. Lower spectrum shows spectrum of CID (collision induced dissociation) of the [M-H]ˉ ion at *m/z* 491.1 ion of malvidin-glucoside. (TIF 41 kb)
Additional file 5: Figure S4. Structural characterization of gallic acid standard by Mass spectra, using MS^n^. Upper spectrum shows full spectrum of gallic acid. Lower spectrum shows spectrum of CID of the [M-H]ˉ ion at *m/z* 168.8 of gallic acid. (TIF 35 kb)
Additional file 6: Figure S5. Structural characterization of quercetin-glucoside standard by Mass spectra, using MS^n^. Upper spectrum shows full spectrum of quercetin-glucoside. Lower spectrum shows spectrum of CID of the [M-H]ˉ ion at *m/z* 463.1 of quercetin-glucoside. (TIF 30 kb)
Additional file 7: Figure S6. Structural characterization of kaempferol standard by Mass spectra, using MS^n^. Upper spectrum shows full spectrum of kaempferol. Lower spectrum shows spectrum of CID of the [M-H]ˉ ion at *m/z* 285 ion of kaempferol. (TIF 32 kb)

## Abbreviations

ABAP: 2,2-azobis(2-methylpropionamide)hydrochloride
CID: Collision induced dissociation
DAPI: 4′,6-diamidino-2-phenylindole
FDA: Fluorescein diacetate
GSC: Green suspension cell cultures
H_2_DCFDA: 2′,7′-dichlorodihydrofluorescein diacetate
HR: Hypersensitive reaction
LC-ESI-MS: Liquid chromatography - electrospray ionization - mass spectrometry
LSD: Least significant difference
MS^n^: Multi-stage mass spectrometry
PBS: Phosphate buffered saline
PCD: Programmed cell death
ROS: Reactive oxygen species
RSC: Red suspension cell cultures
TUNEL assay: *In situ* detection of DNA fragmentation
