# Supplementary figures and images for: Flavonoids and darkness lower PCD in senescing Vitis vinifera suspension cell cultures
Source: BMC Plant Biol. 2016 Oct 26;16:233. doi: 10.1186/s12870-016-0917-y (PMC5080730; doi:10.1186/s12870-016-0917-y)

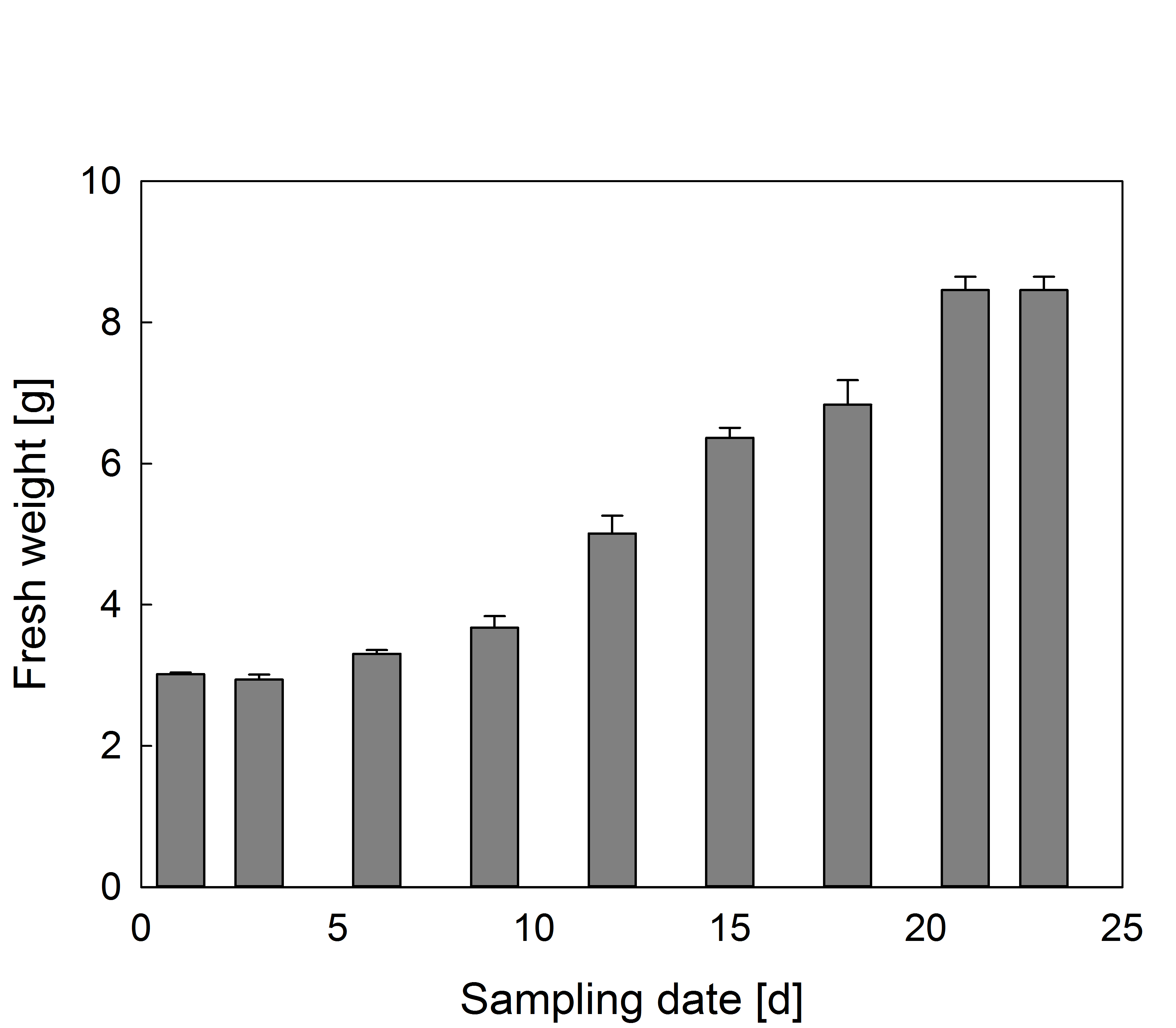

Supplement: Additional file 1: Figure S1. — Proliferation rate of V. vinifera (cv. Limberger) cell cultures grown on solid medium. Cell cultures were grown under light for 23 days. Bars represent means ± S.D. of at least three independent experiments. (TIF 176 kb) [file 12870_2016_917_MOESM1_ESM.tif]

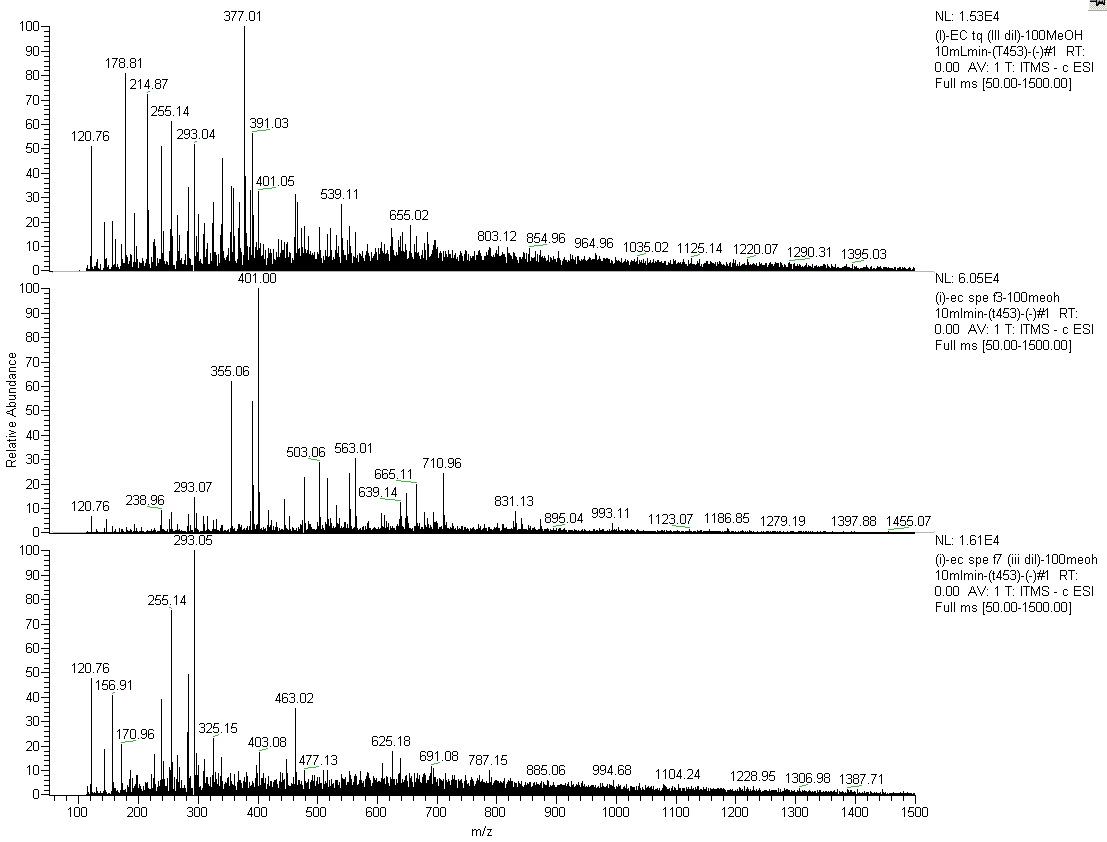

Supplement: Additional file 3: Figure S2. — Mass spectra obtained using Multi-stage Mass Spectrometry (MSn) for analysis of alcoholic extracts obtained by V. vinifera (cv. Limberger) suspension cell cultures. Upper spectrum represents the spectrum obtained from crude extract obtained from RSC grown for 6 days under light. Middle spectrum and lower spectrum represent spectra from two different fractions eluted with H2O and methanol from C18 SPE column, respectively. (TIF 47 kb) [file 12870_2016_917_MOESM3_ESM.tif]

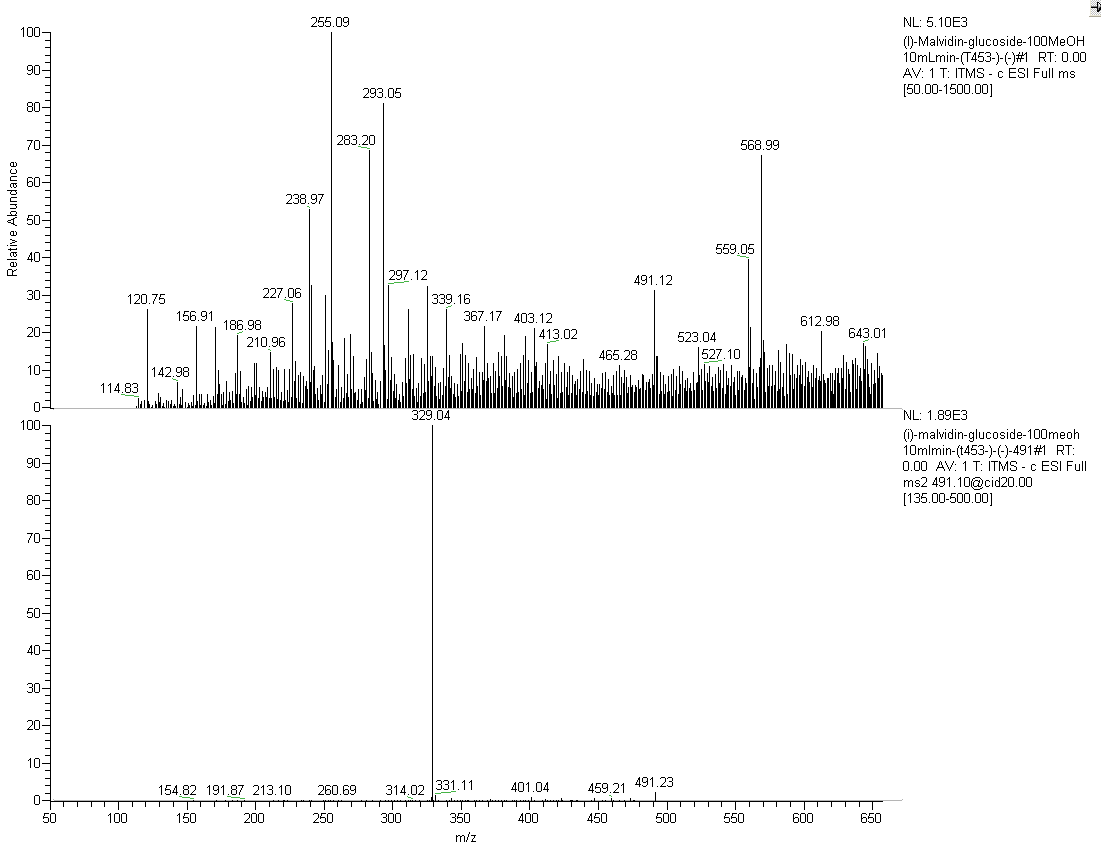

Supplement: Additional file 4: Figure S3. — Structural characterization of malvidin-glucoside standard by Mass spectra, using MSn. Upper spectrum shows full spectrum of malvidin-glucoside. Lower spectrum shows spectrum of CID (collision induced dissociation) of the [M-H]ˉ ion at m/z 491.1 ion of malvidin-glucoside. (TIF 41 kb) [file 12870_2016_917_MOESM4_ESM.tif]

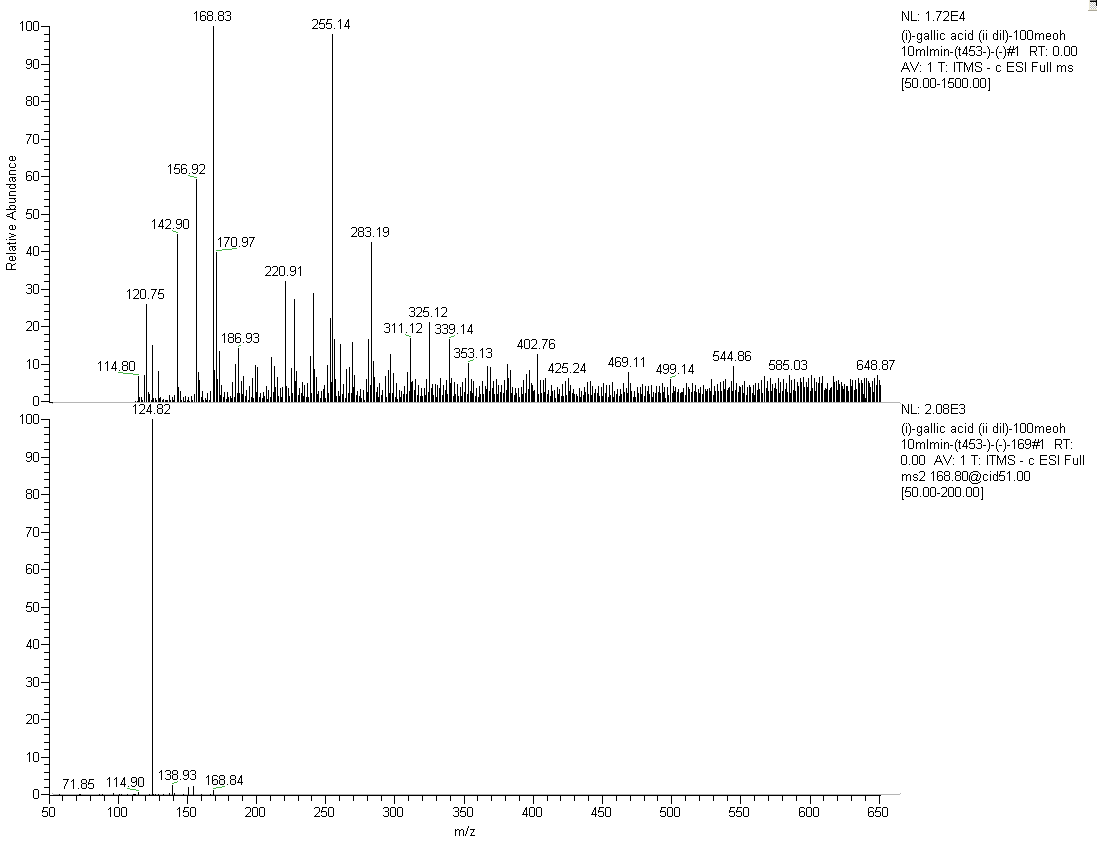

Supplement: Additional file 5: Figure S4. — Structural characterization of gallic acid standard by Mass spectra, using MSn. Upper spectrum shows full spectrum of gallic acid. Lower spectrum shows spectrum of CID of the [M-H]ˉ ion at m/z 168.8 of gallic acid. (TIF 35 kb) [file 12870_2016_917_MOESM5_ESM.tif]

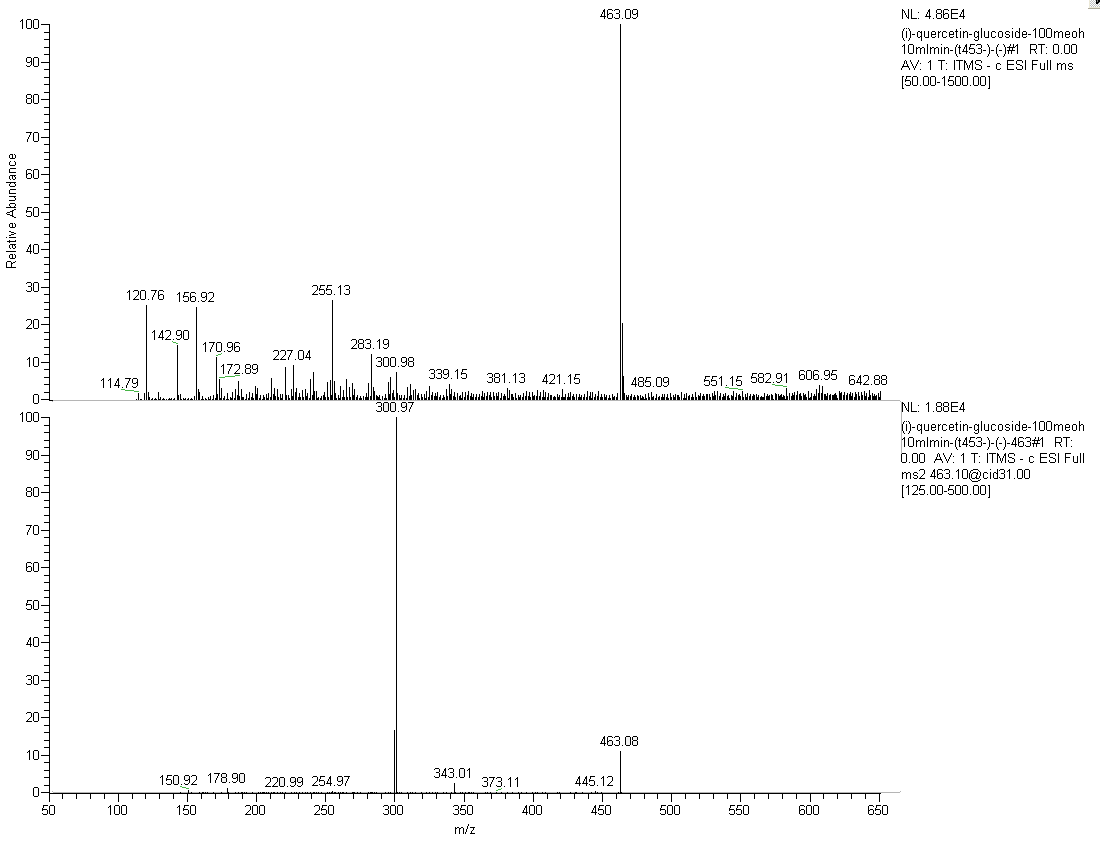

Supplement: Additional file 6: Figure S5. — Structural characterization of quercetin-glucoside standard by Mass spectra, using MSn. Upper spectrum shows full spectrum of quercetin-glucoside. Lower spectrum shows spectrum of CID of the [M-H]ˉ ion at m/z 463.1 of quercetin-glucoside. (TIF 30 kb) [file 12870_2016_917_MOESM6_ESM.tif]

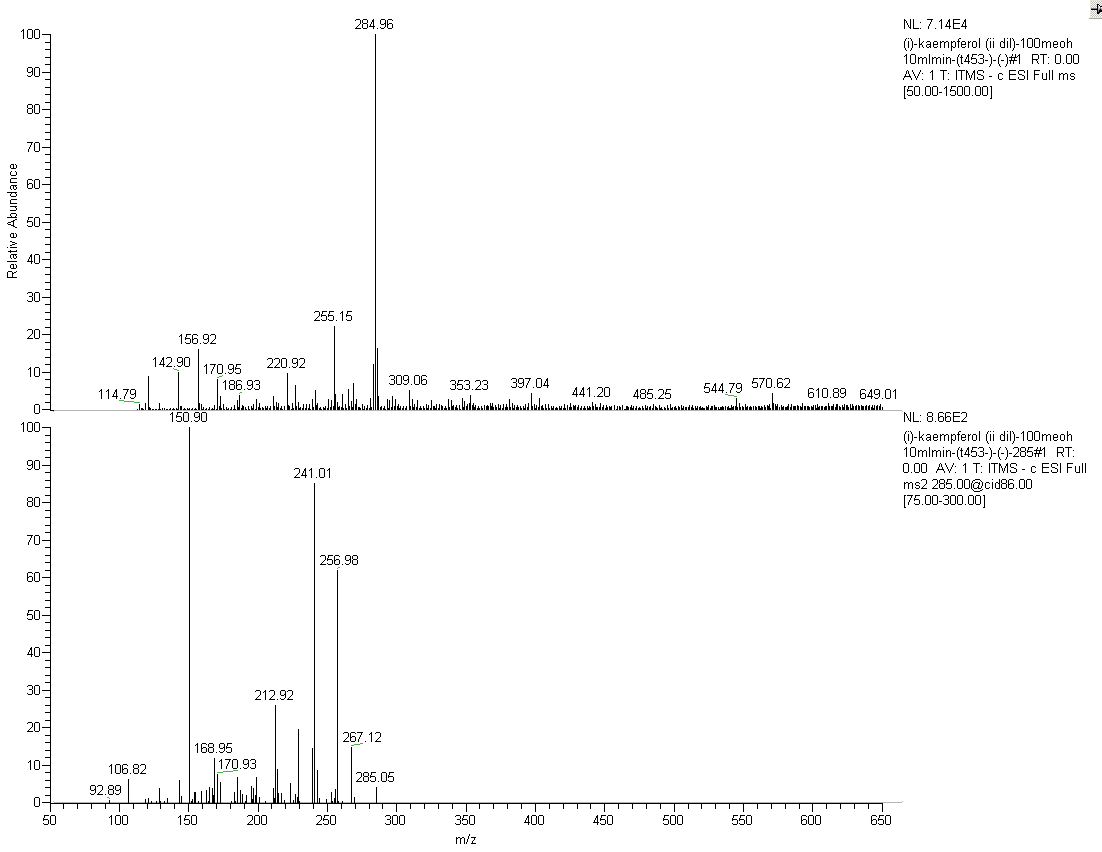

Supplement: Additional file 7: Figure S6. — Structural characterization of kaempferol standard by Mass spectra, using MSn. Upper spectrum shows full spectrum of kaempferol. Lower spectrum shows spectrum of CID of the [M-H]ˉ ion at m/z 285 ion of kaempferol. (TIF 32 kb) [file 12870_2016_917_MOESM7_ESM.tif]
