# Supplementary material for: Flavonoids and darkness lower PCD in senescing Vitis vinifera suspension cell cultures
Source: BMC Plant Biol. 2016 Oct 26;16:233. doi: 10.1186/s12870-016-0917-y (PMC5080730; doi:10.1186/s12870-016-0917-y)
Supplement: Additional file 2: Table S1. — Senescence hallmarks in GSC and RSC. Measurement of total protein content and hexose concentration in GSC and RSC at day 0, just before liquid culture establishment. (DOCX 13 kb) [file 12870_2016_917_MOESM2_ESM.docx]

**Table IS. Senescence hallmarks in GSC and RSC.** Measurement of total protein content and hexose concentration in GSC and RSC at day 0, just before liquid culture establishment.

|  | Total protein | Glucose | Fructose |
| --- | --- | --- | --- |
|  | mg prot ∙ g^-1^ FW | µmol ∙ g^-1^ FW | µmol ∙ g^-1^ FW |
| GSC | 36.35 ± 6.26 | 38.06 ± 1.86 | 7.71 ± 1.39 |
| RSC | 19.71 ± 1.19 | 37.17 ± 1.62 | 6.80 ± 2.19 |
| *t*-test | 0.01 | 0.57 | 0.58 |
